# A Polypill for primary prevention of cardiovascular disease: A feasibility study of the World Health Organization

**DOI:** 10.1186/1745-6215-12-3

**Published:** 2011-01-05

**Authors:** Elsayed Z Soliman, Shanthi Mendis, Wasantha P Dissanayake, Noel P Somasundaram, Padma S Gunaratne, I Kumudini Jayasingne, Curt D Furberg

**Affiliations:** 1 Division of Public Health Sciences, Wake Forest University School of Medicine, Winston-Salem, NC, USA; 2 Department of Chronic Diseases and Health Promotion (CHP), World Health Organization, Geneva, Switzerland; 3 Teaching (General) Hospital Kegalle, Ministry of Health, Sri Lanka; 4 The National Hospital of Sri Lanka, Ministry of Health, Sri Lanka; 5 Teaching (General) Hospital Kandy, Ministry of Health, Sri Lanka

## Abstract

**Background:**

The feasibility of conducting a large-scale Polypill clinical trial in developing countries remains unclear. More information is needed regarding the efficacy in reducing the risk factors of cardiovascular disease (CVD), side effects, improvement in adherence and physician/patient "acceptability" of the Polypill.

**Methods:**

We conducted an open-label, parallel-group, randomized clinical trial involving three sites in Sri Lanka that enrolled a total of 216 patients without established CVD. The trial compared a Polypill (75 mg aspirin, 20 mg simvastatin, 10 mg lisinopril and 12.5 mg hydrochlorothiazide) to Standard Practice. After randomization, patients were followed monthly for three months. Pre-specified primary outcomes included reduction in systolic blood pressure, total cholesterol and estimated 10-year CVD risk. We also evaluated the recruitment process and acceptability of the Polypill by both physicians and patients.

**Results:**

Patients were recruited in a six-month period as planned. Two hundred three patients (94.0%) completed the treatment program and returned for their three-month follow-up visits. No safety concerns were reported. These findings suggest a high rate of patient acceptability, a finding that is bolstered by the majority of patients completing the trial (90%) indicating that they would take the Polypill "for life" if proven to be effective in reducing CVD risk. Approximately 86% of the physicians surveyed agreed with and supported use of the Polypill for primary prevention and 93% for secondary prevention of CVD. Both the Polypill and Standard Practice resulted in marked reductions in systolic blood pressure, total cholesterol and 10-year risk of CVD. However, the differences between the treatment groups were not statistically significant.

**Conclusions:**

We successfully completed a Polypill feasibility trial in Sri Lanka. We were able to document high acceptability of the Polypill to patients and physicians. We were unable to estimate the risk factor reductions on the Polypill because the control group received similar treatment with individual drugs. The Polypill was however simpler and achieved comparable risk factor reductions, highlighting its potential usefulness in the prevention of CVD.

**Trial registration number:**

ISRCTN: NCT00567307

## Background

It has been well documented that aspirin [1], statins [2] and antihypertensive interventions [3] reduce the risk of cardiovascular disease (CVD). These benefits apply both to patients with established CVD (secondary prevention) and those at high risk of developing CVD (primary prevention). Because these interventions exert their positive effects through different mechanisms of action, one might expect that their combined effects to be additive. Wald and Law [4] introduced the concept of a so-called Polypill, a combination of these treatments plus folic acid. Even by using lower doses of the components, they estimated an overall 80% reduction in CVD risk with the Polypill. A recently published primary prevention trial of a Polypill, the double-blinded Indian Polycap Study (TIPS), suggested that the overall benefit might be closer to 60% [5]. The difference between the TIPS trial estimates and the Wald and Law predictions can be explained by the lower dose of simvastatin used in the TIPS trial (20 mg rather than 40 mg) together with non-adherence to treatment [6].

Prior to investing in large-scale, long-term, primary prevention trials designed to determine CVD risk reduction with a fixed dose combination pill in low resource settings several questions regarding feasibility need to be addressed. Would physicians and patients accept the long-term use of a fixed-dose combination pill? Would the components of a Polypill cause side effects? Would a Polypill improve adherence of patients? Would a Polypill reduce CVD risk factors such as high cholesterol or raised blood pressure and decrease CVD risk better than standard treatment?

To address these questions and other relevant issues such as study feasibility, we conducted a clinical trial sponsored by the World Health Organization (WHO) in Sri Lanka. The primary objectives of the trial included estimation of CVD risk reduction and evaluation of side effects of the Polypill and participant adherence to the study intervention. Secondary aims focused on physician and patient "acceptability" of the Polypill and feasibility of the recruitment process.

## Methods

### Study design and population

We conducted an open-label, parallel-group, randomized clinical trial comparing a Polypill to Standard Practice (defined as usual care administered to patients with similar conditions). From February 2, 2009 through July 22, 2009, 591 patients were screened in three clinical sites in Sri Lanka (The National Hospital of Sri Lanka, Colombo; Teaching Hospital, Kegalle, and Teaching Hospital, Kandy). Of the screened patients, 216 (36.5%) were enrolled and randomized, 105 to the Polypill and 111 to the standard practice arm. Patients were eligible for the study if they:

• Were ≥ 50 years old if female and ≥ 40 years if male;

• Had an estimated 10-year total CVD risk score ≥ 20%, based on country-specific WHO CVD risk prediction charts [7]; and

• Had no contraindications to any of the components included in the Polypill.

We excluded those with established CVD, advanced kidney or liver diseases, other life-threatening diseases and those who were unwilling to sign the informed consent.

### Intervention

A Polypill (Red Heart pill 2b, *Reddy's Laboratories, India*) containing 75 mg aspirin, 20 mg simvastatin, 10 mg lisinopril and 12.5 mg hydrochlorothiazide was compared to the level of Standard Practice as defined by the study investigators. Decisions related to discontinuing the study interventions or adding medications during study participation were made at the discretion of the specialist physicians who were in charge of patient care.

### Study flow

Subsequent to granting written informed consent, patients underwent screening and baseline evaluations to confirm eligibility, followed by randomization to the Polypill or to the Standard Practice study arm. Patients receiving either intervention returned for a total of three monthly clinic visits. Data collected included the following:

• Specific questions about anticipated side effects (monthly);

• General inquiry about other complaints (monthly);

• Adherence to study medication via pill count and self report (monthly);

• Fasting levels of total cholesterol, blood sugar, creatinine, potassium and liver enzymes (baseline and 3 month visits)

• Estimated 10-year CVD total risk score using the WHO CVD prediction chart [7]; the score is based on systolic blood pressure and total cholesterol measures as well as on medical history data (monthly);

• "Acceptability" of the Polypill (evaluated in all patients who completed the study [N = 203] as well as in a sampling of screened but ineligible patients (N = 207).

### Physician survey

A five-question survey and the Wald and Law article describing the concept of a Polypill [4] were mailed to 84 physicians from the participating clinical sites and to the Council of General Practitioners in Sri Lanka. All physicians subsequently received reminder phone calls about completing the survey. A total of 58 (69%) physicians (23 internists, 22 general practitioners, 11 cardiologists and 2 others) returned the survey. Physicians were asked about their prior knowledge of the Polypill concept, their agreement with Wald and Law that there is no need to monitor CVD risk factors, and their opinion about which factors would most affect their decision to prescribe the Polypill. Finally, they were asked about their acceptability in prescribing the Polypill for purposes of primary and secondary prevention of CVD.

### Statistical analysis

In estimating the sample size for our study, an assumption was made that 200 patients would be needed to achieve > 80% power at alpha = 0.05 in order to detect a 6 mmHg difference in systolic blood pressure, a 12 mg/dl (0.31 mmol/L) difference in total cholesterol and an approximate 3-5% absolute difference in the estimated 10-year CVD risk between the two study groups. The standard deviations around the mean baseline values were assumed to be 14 mmHg and 20 mg/dl for systolic blood pressure and total cholesterol, respectively.

Frequency distributions for all variables were first inspected to identify outliers possibly caused by measurement errors. Descriptive statistics were presented as means with standard deviations (SD) for all study participants, stratified by intervention arm at baseline. Student T and Chi square tests were employed to compare changes in outcome variables between intervention arms. All comparisons were made on an intention-to-treat basis. A two-tailed p < 0.05 was considered significant at alpha level of 0.05. SAS, version 9.1 (SAS Institute, Inc., Cary, North Carolina) was used in all analyses.

### Ethical approval and registration

The World Health Organization Research Ethics Review Committee, the Institutional Review Board of Wake Forest University Health Sciences, and Sri Lanka Ministry of Health approved this clinical trial. The trial is registered with ClinicalTrials.gov protocol registration system, ID NCT00567307.

## Results

Table 1 summarizes the characteristics of the study population stratified by intervention assignment. Baseline characteristics were generally well balanced across the two study groups, with Polypill subjects exhibiting a slightly higher level of total cholesterol compared to patients in the Standard Practice group (P = 0.05).

**Table 1:** Baseline characteristics by treatment assignment

	Overall (N = 216)	Standard Practice (N = 111)	Polypill (N = 105)
Gender (female)	157 (72.7%)	77 (69.4%)	80 (76.2%)
Age	59.1 ± 7.2	59.2 ± 7.4	59.0 ± 6.9
Smoking	11.2%	12.6%	9.7%
Estimated 10- year CVD risk			
10 - 19%	2 (0.9%)	0 (0.0%)	2 (1.9%)
20 - 29%	81 (38.2%)	48 (44.4%)	33 (31.7%)
30 - 39%	58 (27.4%)	27 (25.0%)	31 (29.8%)
40% or higher	71 (33.5%)	33 (30.6%)	38 (36.5%)
Systolic blood pressure (mmHg)*	165.2 ± 18.2	164.7 ± 17.3	165.7 ± 19.2
Total cholesterol (mmol/L)	5.9 ± 1.3	5.7 ± 1.3	6.1 ± 1.3
Fasting glucose (mmol/L)	7.1 ± 2.7	7.1 ± 2.6	7.2 ± 2.8
Creatinine (mmol/L)	89.0 ± 21.8	90.4 ± 21.3	87.6 ± 22.3
Serum potassium (mmol/L)	4.6 ± 0.6	4.6 ± 0.6	4.5 ± 0.5
ALT (U/L)	31.2 ± 16.7	32.0 ± 18.1	30.5 ± 15.1
Weight (kg)	57.2 ± 9.4	56.7 ± 9.2	57.8 ± 9.6
Height (cm)	152.8 ± 9.2	153.3 ± 9.2	152.3 ± 9.3

* Second measured blood pressure

Table 2 summarizes the post-randomization use of non-study antihypertensive drugs and statins. Many more patients in the Standard Practice group were taking these added medications compared to those in the Polypill group (P < 0.01). The difference in use of antihypertensive therapy was 89% vs 0% at one site and 40% vs 5% at another, respectively. Use at the third site was very high for both groups, 94% vs 90%. Trends for post-randomization use of statins were similar, but less pronounced.

**Table 2:** Post-randomization use of non-study antihypertensive drugs and statins

Site	Medications	Standard Practice	Polypill	P-value
Kegalle Teaching Hospital	Antihypertensive use	8/20 (40.0%)	1/21 (4.8%)	< 0.01
	Statin use	5/20 (25.0%)	0/21 (0.0%)	0.01

Kandy Teaching Hospital	Antihypertensive use	33/37 (89.2%)	0/34 (0.0%)	< 0.01
	Statin use	29/37 (78.4%)	2/30 (6.7%)	< 0.01

The National Hospital of Sri Lanka	Antihypertensive use	51/54 (94.4%)	45/50 (90.0%)	0.40
	Statin use	41/54 (75.9%)	35/50 (70.0%)	0.50

All sites (Total Sample)	Antihypertensive use	92/111 (82.9%)	46/105 (43.8%)	< 0.01
	Statin use	75/111 (67.6%)	37/101 (36.6%)	< 0.01

Approximately 94% (N = 203) of the patients completed the trial (104 in the Standard Practice group and 99 in the Polypill group). Over 80% randomized to the Polypill demonstrated >80% adherence to the pill, with only 10%, 3%, and 6%, reporting <40%, 40-59% and 60-79% adherence, respectively. In those who reported adherence <80% (N = 19), 10 patients attributed their low levels of adherence to side effects, 2 patients said that they forgot to take the pill, and 7 patients did not specify reasons. However, side effects and complaints such as epigastric pain, cough and musculoskeletal pain were comparable between the two study arms (Table 3).

**Table 3:** Cumulative frequency of major side effects during the 3-month treatment period by study group

	Standard Practice (N = 104)	Polypill (N = 99)	Odds ratio (95% CI)	p-value
Epigastric pain	20/104 (19.2%)	15/97 (15.5%)	1.30 (0.62, 2.72)	0.482

Musculoskeletal pain	29/104 (27.9%)	26/97 (26.8%)	1.06 (0.57, 1.96)	0.864

Cough	18/104 (17.3%)	25/97 (25.8%)	0.60 (0.30, 1.19)	0.144

Other symptoms	12/104 (11.5%)	15/97 (15.5%)	0.71 (0.32, 1.61)	0.415

There was no significant difference between the two intervention groups with regard to reduction in estimated 10-year CVD risk, systolic blood pressure, and total cholesterol (Table 4). Other laboratory measures (serum creatinine, potassium, and ALT) were also comparable.

**Table 4:** Change in estimated 10-year CVD risk and key measures at study closeout

	Standard Practice (N = 104)			Polypill (N = 99)				
			
	Baseline	Exit	Diff	Baseline	Exit	Diff	Diff of Differences (95% CI)	P-value
Estimated 10-year CVD risk (%)	41.6 ± 19.8	11.1 ± 10.0	-30.6 ± 22.8	44.1 ± 20.3	11.5 ± 13.0	-32.7 ± 26.0	2.1 (-4.9, 9.1)	0.56
Systolic blood pressure (mmHg)*	165.0 ± 17.6	138.1 ± 19.3	-26.9 ± 25.7	165.6 ± 19.2	136.8 ± 20.8	-28.8 ± 24.9	1.9 (-5.1, 8.9)	0.60
Total cholesterol (mmol/L)	5.7 ± 1.3	4.7 ± 1.1	-1.0 ± 1.6	6.1 ± 1.3	4.8 ± 1.0	-1.4 ± 1.2	0.4 (0.0, 0.8)	0.07
Creatinine (mmol/L)	90.5 ± 21.2	92.8 ± 24.6	1.3 ± 19.3	87.8 ± 22.6	92.9 ± 25.9	4.8 ± 31.7	-3.5 (-10.8, 3.8)	0.36
Serum potassium (mmol/L)	4.6 ± 0.6	4.5 ± 0.4	-0.0 ± 0.5	4.5 ± 0.5	4.6 ± 0.5	0.1 ± 0.6	-0.1 (-0.3, 0.1)	0.10
Alanine aminotransferase (U/L)	31.9 ± 18.2	36.3 ± 31.6	4.1 ± 25.7	29.8 ± 15.0	33.0 ± 26.0	3.0 ± 17.8	1.1(-5.1, 7.3)	0.72

* Second measured blood pressure

### Patient acceptability

Approximately 90% of the patients completing the study (93% in the Standard Practice group and 86% in the Polypill group) indicated that they would "definitely" or "probably" take the pill for life if it were shown to be effective in reducing CVD risk. A similar response level (89%) was obtained from those who were screened but found to be ineligible for the study.

### Physician acceptability

Physicians were fairly aware of the concept of the Polypill (Table 5**)**. Although they seemed skeptical about not to monitor CVD risk factors (as suggested by Wald and Law), they demonstrated high acceptability for prescribing the Polypill as a therapeutic tool for both primary prevention (86%) and secondary prevention (93%). Reduction in total 10-year CVD risk appeared to be the most important factor that would factor into a decision to prescribe the pill. These results did not vary by specialty.

**Table 5:** Physician survey results

Question	Summary response
Q1: Knowledge of the Polypill concept	
It has been hypothesized through a meta-analysis that a daily intake of a so-called Polypill (fixed combination of low dose ACE-I, statin, diuretic and aspirin) for life long may reduce the risk of CVD by more than 80%. These results, according to the hypothesis, are applicable to primary and secondary prevention. In a scale from 1 to 10 where 1 is the least and 10 is the maximum, how do you rate your knowledge about the Polypill?	*Mean (SD); Median (Minimum, Maximum)* 5.6 (2.2); 6.0 (1.0, 10.0)

Q2: Agreement with Wald & Law on lack of need to routinely monitor CVD risk factors	
The polypill strategy, as suggested by the authors, does not require routine monitoring or measuring the cardiovascular risk factors (such as blood pressure or cholesterol) in patients receiving the Polypill. This has been rationalized by the notion that CVD risk reduction by the Polypill is independent from the baseline value. In a scale from 1 to 10 where 1 is the least and 10 is the maximum, how do you rate your agreement on not to monitor the CVD risk factors in patients receiving the Polypill?	*Mean (SD); Median (Minimum, Maximum)* 2.9 (2.4); 2.0(1.0, 10.0)

Q3: Most important factor for prescribing a Polypill	
What would be the most important factor that determine your decision to prescribe the Polypill:	
	*N (%)*
Few side effects	11(19.3)
Degree of CVD risk reduction	28 (49.1)
Low cost	5 (8.7)
Others	14 (24.6)

Q4: Acceptability to prescribe the Polypill for PRIMARY CVD prevention	
If it is documented in a large clinical trial that a daily intake of the Polypill reduces the risk of major CVD in people without established CVD (*primary prevention***)**, will you prescribe it to your patients?	
	*N (%)*
Yes, definitely	39(67.2)
Yes, probably	11(19.0)
No	8 (13.8)

Q5: Acceptability to prescribe the Polypill for SECONDARY CVD prevention	
If it is documented in a large clinical trial that a daily intake of the Polypill reduces the risk of recurrent cardiovascular event in people with established CVD (*secondary prevention*) will you prescribe it to your patients?	
	*N, (%)*
Yes, definitely	50 (86.2)
Yes, probably	4 (6.9)
No	4(6.9)

## Discussion

### Overall findings

The feasibility trial met several of our goals. Enrollment of study patients went very well and was completed according to the protocol-specified schedule. Most patients in the Polypill group adhered to the study medications. Only 6% of the enrollees did not complete the treatment program and returned for the 3-month visit. There were no reported safety concerns. These findings are consistent with a high rate of patient acceptability. The majority of patients completing the trial expressed support for taking the Polypill for life if it were shown to be effective in reducing CVD risk. Similarly, 86% of physicians surveyed agreed with and supported the use of the Polypill for primary prevention and 93% for secondary prevention of cardiovascular disease.

The double-blinded Indian Polycap Study (TIPS) evaluated the effects of several drug combinations on blood pressure and lipids over 12 weeks. The Polycap reduced systolic blood pressure by 7.4 mmHg and LDL cholesterol by 0.70 mmol/L compared to no treatment. TIPS excluded subjects who were taking any component drugs included in the Polycap [5].

In contrast to TIPS, we did not detect any differences between the Polypill and Standard Practice groups in terms of reductions in systolic blood pressure, total cholesterol or 10-year risk of cardiovascular disease. This could relate to a number of problems that were revealed during data analysis. It appears that the Standard Practice group received an unusually high level of care after randomization, which, in turn, raised this study group's level of risk factor intervention to the level of the Polypill group. The marked risk factor reductions observed in both groups can possibly be explained by regression to the mean. The large changes in blood pressures between baseline and follow-up visits also raise questions about the standardization of these measurements.

### Lessons learned

The centers involved in our study were tertiary care hospitals. They do not reflect the level of medical care provided at primary care centers and secondary level hospitals in Sri Lanka. Thus, the findings from the selected trial sites cannot be generalized to other levels of care.

Furthermore, there is evidence that special attention was given to Standard Practice patients. At two sites, a much higher proportion of them were prescribed anti-hypertensive drugs and statins compared to those in the Polypill group. At the third site, a large proportion of enrolled patients received antihypertensive therapy and statins. Regardless of the details of treatment prescribed, which would be of limited value here, this use of non-study drugs defeated the objective of the trial by making it difficult to observe the expected treatment effect of the Polypill. Clearly the open-label design of our study undermined our ability to properly test the Polypill.

The extent of dose adjustments made in the Standard Practice group during the 3-month treatment period is not known. Such adjustments could have further diminished the study group differences for systolic blood pressure and total cholesterol.

Based on the challenges we encountered in our trial, we think that there are four design options to consider for future Polypill studies. First, would be to conduct the trial on people above 50 years of age and not focus on risk factor measurement. Second, would be to follow the suggestion by Wald and Law [4] and not monitor the risk factors. Third, would be to recruit a low risk population in which treatment of risk factors would be much less prevalent. Fourth, would be to strongly discourage the investigators to alter any risk factor treatment after randomization

## Conclusions

We successfully completed a Polypill feasibility trial in Sri Lanka. We were able to document high acceptability of the Polypill to patients and physicians. We were unable to estimate the risk factor reductions on the Polypill because the control group received similar treatment with individual drugs. The Polypill was however simpler and achieved comparable risk factor reductions, highlighting its potential usefulness in the prevention of CVD. Further studies assessing the Polypill in developing countries should take into consideration the study design lessons and challenges that we encountered.

## Competing interests

The authors declare that they have no competing interests.

## Authors' contributions

EZS, CDF and SM conceived the study and participated in its design and coordination and drafted the manuscript. WPD, NPS, PSG and IKJ participated in the acquisition of data and obtained local ethics approval. All authors read and approved the final manuscript.
